# An intramedullary Echidna pin for fixation of comminuted clavicle fractures: a biomechanical study

**DOI:** 10.1186/s13018-017-0623-y

**Published:** 2017-08-11

**Authors:** David Ackland, Ian Griggs, Patrick Hislop, Wen Wu, Minoo Patel, Martin Richardson

**Affiliations:** 1 Department of Biomedical Engineering, University of Melbourne, Parkville, Victoria 3010 Australia; 2 Mooroolbark Veterinary Clinic, Moorolbark, Victoria 3138 Australia; 3 Department of Surgery, University of Melbourne, Epworth Healthcare, Richmond, Victoria 3121 Australia; 4 Centre for Limb Reconstruction, The Epworth Centre, Richmond, Victoria 3121 Australia; 5 Department of Surgery, Southern Clinical School, Monash University, Clayton, Victoria 3168 Australia

**Keywords:** Repair construct, Biomechanics, Surgery, Plate, Screw, Shoulder, Upper limb

## Abstract

**Background:**

Intramedullary fixation of comminuted mid-shaft clavicle fractures has traditionally been employed with satisfactory clinical outcomes; however, pins with smooth surfaces may protrude from the bone and are prone to migration, while some threaded pins are difficult to remove post-operatively. The aim of this proof-of-concept study was to develop and evaluate the biomechanical strength of a novel intramedullary Echidna pin device designed to maintain fracture reduction, resist migration and facilitate ease of post-operative removal.

**Methods:**

Thirty human clavicle specimens were harvested and fractured in a comminuted mid-shaft butterfly configuration. Each specimen was randomly allocated to three surgical repair groups including intramedullary fixation using the Echidna pin and Herbert Cannulated Bone Screw System, as well as plate fixation using bi-cortical locking screws. Using a biomechanical testing apparatus, construct bending and torsional stiffness were measured, as well as ultimate bending strength.

**Results:**

There was no significant difference in torsional stiffness and ultimate bending moment between the Echidna pin and Herbert screw repair constructs (*p* > 0.05); however, the Echidna pin construct demonstrated a significantly greater bending stiffness compared to that of the Herbert screw construct (mean difference 0.55 Nm/deg., *p* = 0.001). The plate construct demonstrated significantly greater torsional stiffness, bending stiffness and ultimate bending moment compared to those of the Herbert screw and Echidna pin (*p* < 0.05).

**Conclusions:**

An intramedullary Echidna pin device was designed to stabilize comminuted fractures of the clavicle, maintain fracture compression and provide ease of removal post-operatively. Since the results suggest equivalent or superior torsional and bending stability in the Echidna pin compared to that of the Herbert screw, the Echidna pin concept may represent an alternative fixation device to conventional intramedullary screws, nails and pins; however, superior plating using bi-cortical locking screws provides substantially higher construct structural rigidity than intramedullary devices, and may therefore be useful in cases of osteoporotic bone, or where high fracture stability is required.

## Background

Middle-third clavicle fractures represent approximately 80% of all clavicle fractures, with over half of these fractures displaced [1, 2]. Fracture displacement of 20 mm, shortening, as well as comminution, has been shown to increase risk of persistent symptoms and poor functional outcome [3]. The conservative treatment of displaced mid-shaft fractures of the clavicle has been shown to result in unsatisfactory outcomes in 30% of patients, including malunion, poor cosmetic results and loss of upper limb strength, with moderate pain as the most commonly reported complication [4–6]. As a consequence, surgical treatment has become the standard of care for displaced fractures, and has been shown to result in increased patient satisfaction at up to 1 year follow-up [7].

Indications for surgical treatment of acute mid-third clavicle fractures include non-union with concomitant pain, open fracture, severe malpositioning, skin tenting and a fracture gap greater than half of one clavicle diameter [8]. Open reduction followed by fixation using a plate or intramedullary device represent the most commonly employed surgical approaches for treatment of displaced mid-third fractures.

Plate fixation includes the use of dynamic compression plates, tubular plates or reconstruction plates [9], and may provide greater construct rigidity, particularly when used with bi-cortical locking screws; however, plate fixation has been shown to present risk of stress shielding at the fracture site, re-fracture after implant removal, hypertrophic scars and, in rare cases, subclavian vessel or brachial plexus damage resulting from over-drilling or excessive bi-cortical screw penetration [10–13]. Intramedullary devices such as the Rockwood pin, Herbert screw, Titanium Elastic Nail, Kirschner wires and Knowles pin preserve the soft tissue envelope and vascular structures, require smaller surgical wounds than plate fixation, reduce the likelihood of infection and improve callus formation [12, 14, 15]. While some devices may be removed under local anaesthesia using a 2- to 3-cm incision [15, 16] and have resulted in excellent cosmetic outcome and low non-union rates, intramedullary fixation can be technically demanding [13]. A number of fixation devices have also been associated with pin migration through the skin, aorta, lung and spinal canal [13, 17, 18].

Kirschner wires, Steinmann pins and Knowles and Hagie pins have been employed with good clinical and function outcomes [8, 19–21]; however, because of their smooth surfaces, pointed ends and varying degrees of pin length protrusion from the bone, they are prone to migration and may provide low initial torsional support [13, 22, 23]. The Herbert cannulated bone screw is a variable-pitch screw, threaded at each end, which can be used to create fracture compression. While this device resides entirely within the bone, it is considered a permanent fixation device and can be challenging to remove [24, 25]. A novel threadless intramedullary Echidna pin device was developed, featuring a series of retractable fixation spines that may be deployed into the cancellous and cortical bone intraoperatively to maintain fracture reduction, provide construct axial and torsional stability and ultimately facilitate ease of post-operative removal. The aim of this proof-of-concept study was to employ a comminuted mid-shaft clavicle fracture model to evaluate the torsional and bending stiffness as well as the ultimate strength of the Echidna pin repair construct, and compare the results to those of the Herbert screw and a gold-standard superior plating. We hypothesise that the Echidna pin repair construct will exhibit comparable bending and torsional stiffness to that of the Herbert screw construct, and that both the Echidna pin and Herbert screw constructs will exhibit lower stiffness and bending strength compared to that of the plate construct.

## Methods

### Specimen preparation

Thirty embalmed clavicle specimens (13 left, 17 right; mean age 85.1 years, range 62 to 91 years) were harvested from 17 cadavers. Based on previously reported biomechanical studies evaluating fixation strength of mid-shaft fracture repair constructs, we proposed a sample size of 30, with 10 specimens per group to obtain a statistical power of 0.8 for a bending stiffness effect [26, 27]. All soft tissue was removed, and specimens were radiographed with X-ray fluoroscopy to screen for fracture or osseous abnormalities. Ethical approval for this study was obtained through the University of Melbourne Departmental Human Ethics Advisory Group (#1441640), and participants’ next of kin provided informed consent.

### Clavicle fracture and surgical repair

A mid-shaft osteotomy was first made on the middle third of each specimen to simulate a ‘B-type’ wedge segmental fracture. A template was used to outline a standardized 20-mm-long and 8-mm-wide diamond shape on the inferior surface of the clavicle in line with the mid-shaft of the clavicle. Using an oscillating bone saw, a wedge-shaped osteotomy was performed by removing the portion of outlined bone to a depth of 3 mm. A transverse mid-shaft cut through the entire clavicle was then performed.

**Fig. 1:**
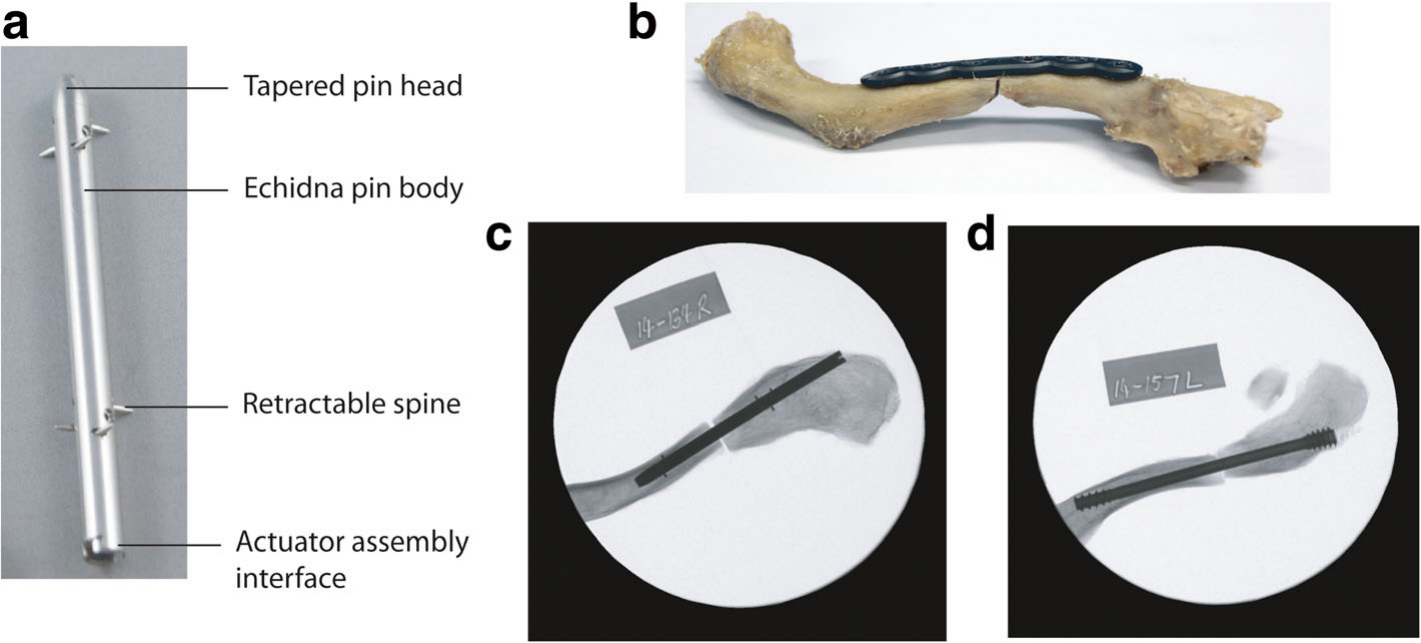
Echidna pin design illustrating body, retractable nails, retractable pin head and actuator assembly interface (**a**). Also shown are mid-shaft fracture repair constructs including **b** superior plating using bi-cortical locking screws, **c** Echidna pin and **d** Herbert screw

Each clavicle was then randomly assigned to one of three surgical repair groups which included intramedullary fixation using an Echidna pin and Herbert screw (Zimmer-Biomet, Warsaw, USA) [24, 25], and plate fixation using a six-hole decreased curvature plate secured with bi-cortical locking screws (VariAx, Stryker, Michigan, USA) [28] (Fig. 1). The Echidna pin was manufactured from 316LVM stainless steel in two standard diameters (5.0 and 6.5 mm) and six lengths (60, 70, 80, 90, 100, and 110 mm). It was deployed into the intramedullary canal using a well-described method [8] without application of torsion. Using a mechanical actuator attached to the medial end of the clavicle, the Echidna pin fixation spines were extended into the surrounding bone, with compression at the fracture site achieved by manual compression of both fragments of the fracture. The Echidna pins and Herbert screws were chosen based on radiographic bone-size measurements, and placed according to the manufacturer specifications. Plates were chosen such that the largest plate width would contour the cadaveric specimens without hardware prominence beyond the cortical borders. The titanium fixation screws used were 3.5 mm in diameter and varied in length between 10 and 24 mm.

### Biomechanical testing

**Fig. 2:**
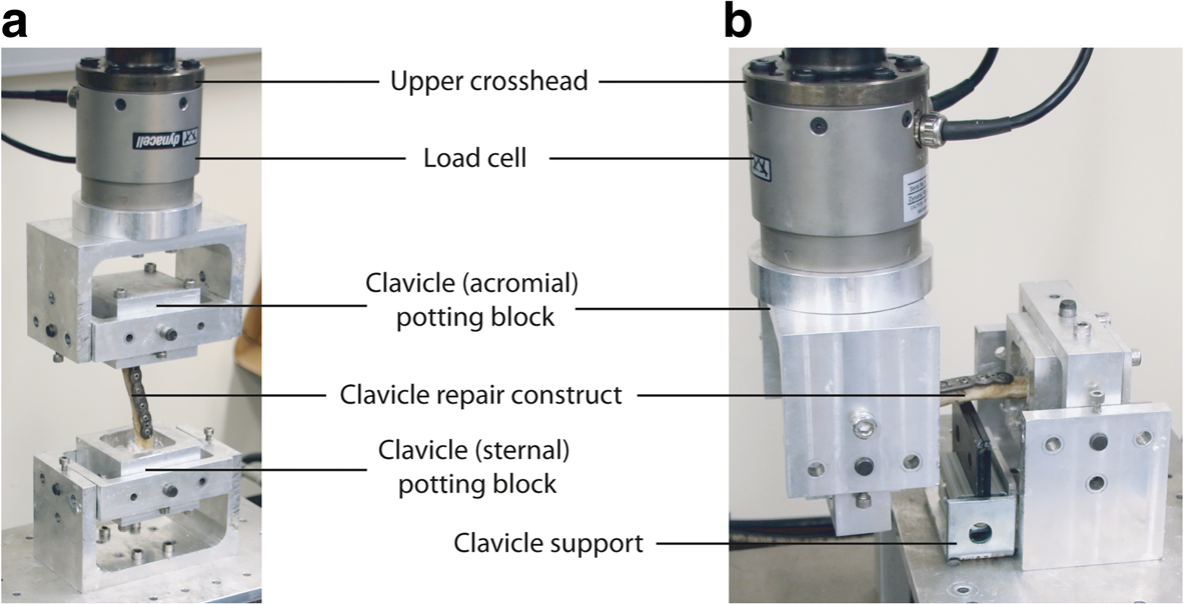
Photographs of clavicle load testing experiments. The clavicle repair constructs were mounted by embedding the sternal and acromial ends of the clavicle in customized potting blocks. The sternal potting block was fastened to the base of the Instron test cell, while the acromial potting block was mounted to a load cell on the upper crosshead of the Instron. Torsion was applied by rotating the acromial potting block with the clavicle oriented vertically (**a**), while bending was applied by displacing the acromial potting block downward with the clavicle oriented horizontally (**b**)

The testing protocol employed was based on a previously reported procedure [28]. All repair constructs underwent non-destructive torsional loading followed by bending to failure. Each clavicle was embedded in customized potting fixtures using dental cement and mounted to an Instron Materials Testing Machine (Instron, Model 3521, Parker Hydraulics) (Fig. 2). The mechanical axis of the clavicle was aligned with the direction of the Instron load actuator. Twisting was applied to the acromial end of the clavicle at a rate of 0.5°/s [29, 30] until a rotation of 3° was reached, whereby the rotation was stopped and 3° of rotation applied in the opposite direction to unload the specimen, followed by an additional 3° of rotation in this same direction. The chosen range of rotations limited the disruption to the repair construct, while ensuring sufficient torque-angle data to calculate angular stiffness. Angular rotation and applied torque were sampled at 25 Hz, and torsional stiffness (Nm/degrees) was calculated from the gradient of the linear regression ‘best-fit’ line applied to the resultant torque vs. rotation curve.

Each repair construct was then loaded in cantilever bending following the torsional testing. To orient the specimen horizontally, the acromial potting fixture was released from the upper Instron crosshead, while the sternal potting fixture was rotated by 90°. A vertical support was placed just medial to the clavicle fracture, to ensure that peak bending moments occurred at the fracture site and not at the sternal end of the clavicle. The acromial end of the clavicle was displaced downward at a constant rate of 0.5 mm/s by lowering the upper crosshead of the Instron [29, 30]. Loading was stopped when the ultimate (maximum) bending moment was achieved. The applied bending moment on the clavicle was calculated by multiplying the applied force magnitude with the force lever arm, which was measured using digital callipers and assumed to be constant. During loading, applied force and displacement of the upper crosshead were sampled at 25 Hz. Angular displacement of the clavicle during bending was calculated trigonometrically using the lever-arm of the applied force and the vertical displacement of the acromial end of the clavicle. The bending stiffness during loading was then calculated from the gradient of the linear regression ‘best fit’ line applied to the bending moment vs. angular displacement curve.

### Statistical analysis

A single-way analysis of variance (ANOVA) was used to evaluate between-group differences in mean bending stiffness, ultimate bending moment and torsional stiffness for the three repair groups, with Games-Howell post hoc tests for unequal variances used to compute mean differences between groups. Standard deviation was used as a measure of the dispersion of results. Level of significance was set at *p* < 0.05. All statistical data analyses were performed using the Statistical Package for the Social Sciences (PASW Statistics 18, SPSS Inc., Chicago, IL).

## Results

### Torsion

**Fig. 3:**
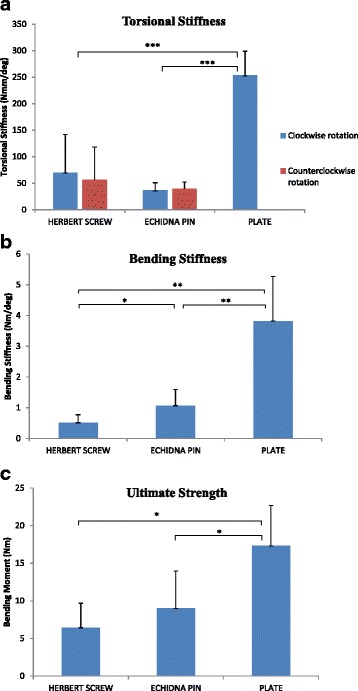
Results of biomechanical testing of comminuted clavicle fracture repair constructs, including those of the Echidna pin, Herbert screw and superior plating. Data given include **a** torsional stiffness, **b** bending stiffness and **c** ultimate bending moment. *Bars* represent mean values, while *whiskers* indicate one standard deviation. *One asterisk* indicates *p* < 0.05, *two asterisks p* < 0.01 and *three asterisks p* < 0.001

Plate constructs were significantly stiffer in torsion than Echidna pin constructs (mean difference 216.9 Nmm/deg, *p* < 0.001) and Herbert screw constructs (mean difference 183.2 Nmm/deg, *p* < 0.001) during clockwise twisting (Fig. 3). While there was a trend of lower torsional stiffness in the Echidna pin construct compared to that in the Herbert screw construct during clockwise (mean difference 33.8 Nmm/deg) and counterclockwise twisting (mean difference 16.4 Nmm/deg), this difference was not significant (*p* > 0.05). The torsional stiffness of the Herbert screw during counter-clockwise twisting was significantly lower than that during clockwise twisting (mean difference 13.9 Nmm/deg, *p* = 0.04), whereas there was no significant direction-dependent torsional stiffness in the Echidna pin construct (mean difference 3.48 Nmm/deg, *p* > 0.05).

### Bending

The plate construct was significantly stiffer in bending than the Echidna pin construct (mean difference 2.76 Nm/deg, *p* < 0.001) and the Herbert screw construct (mean difference 3.31 Nm/deg, *p* < 0.001). The Echidna pin construct demonstrated a significantly greater bending stiffness than that of the Herbert screw construct (mean difference 0.55 Nm/deg, *p* = 0.001). Similar between-group trends were observed in ultimate bending strength, with the plate construct exhibiting a significantly greater ultimate bending moment than that of the Echidna pin construct (mean difference 8.31 Nm, *p* = 0.002) and Herbert screw construct (mean difference 10.9 Nm, *p* = 0.001). While a greater ultimate bending moment was observed in the Echidna pin construct compared to that of the Herbert screw construct (mean difference 2.56 Nm), this difference was not significant (*p* > 0.05).

## Discussion

Open reduction together with internal plate fixation and intramedullary fixation are two of the most common surgical techniques for the treatment of displaced mid-shaft clavicle fractures. A prospective comparison of Knowles pinning and plate fixation suggested that plating has been associated with longer operation time, larger wound incision, higher pain levels, more analgesic use and more symptomatic hardware complications [8]. While intramedullary fixation may result in excellent cosmetic outcome with low non-union rates, devices such as Kirschner wires, Steinmann pins and the Knowles pin may migrate into the surrounding tissue, while multi-threaded devices such as the Herbert screw can be difficult to remove and are usually considered permanent. This proof-of-concept study evaluated the biomechanical performance of a novel intramedullary Echidna pin with retractable spines to engage the surrounding bone and provide torsional stability as well as fracture compression, and facilitate safe and easy post-operative removal. Confirming our hypothesis, there was no significant difference in peak torsional stiffness between the Herbert screw and the Echidna pin. Plating using bi-cortical locking screws outperformed the Echidna pin and Herbert screw constructs in torsion and bending.

Despite significantly lower torsional stiffness, bending stiffness and ultimate bending moment observed in intramedullary fixation constructs compared to those of superior plating, clinical studies suggest no difference in outcome, and even reduced post-operative complication rates compared to that of plate fixation constructs for displaced mid-third clavicle fractures [15, 31, 32]. This may suggest that internal splinting of a fracture site using conventional pins and rods may, with appropriate post-operative limb rehabilitation, result in sufficient bone fixation to allow external bridging callus through intramembranous bone formation [33]. Conversely, the prominent rigidity of repair constructs employing one or more surface plates and bi-cortical screws may have the potential to result in stress shielding and impede the fracture healing process, as modelling and simulation has shown [34]. While the decision to use intramedullary fixation may depend on numerous factors including device cost, surgical time and fracture type, in addition to the practicality of a permanent or migration-prone device, clinical evidence suggests that intramedullary devices can provide sufficient internal fixation to facilitate fracture healing [15], which ought to be considered in light of the complications associated with superior plating, including poor cosmetic outcome, skin irritation and numbness due to nerve damage.

A clinical and radiographic review of symptomatic non-unions of the clavicle treated with open reduction and intramedullary fixation using the Herbert screw revealed satisfactory union with no loosening after 13 months [24]. Since the torsional stiffness of the Echidna pin construct was not significantly different to that of the Herbert screw construct, and the bending stiffness of the Echidna pin construct was significantly higher than that of the Herbert screw construct, the results suggest similar short-term functional performance between the two prostheses. In contrast to the Echidna pin constructs, however, the Herbert screw constructs demonstrated significantly greater torsional stiffness during clockwise loading compared to anti-clockwise loading. Because this device is double threaded, clockwise twisting of the construct had a tendency to compress the fracture site thereby increasing the construct resistance to torsion, whereas anticlockwise motion had a tendency to separate the fracture segments. Since abduction of the upper limb is known to rotate the clavicle about its longitudinal axis by up to 50° [35], the results of the present study highlight the importance of early sling use and restricted elevation of the upper limb immediately after clavicle fracture repair using the Herbert screw.

One shortcoming of clavicular fracture surgery is the requirement for implants to be removed in a second operation [16, 36], which has been shown to be necessary more often in plate fixation than intramedullary fixation [12]. In particular, plates are electively removed in professional athletes who engage in high-contact sports to avoid the difficult management associated with fracture around the plate [15]. Intramedullary devices may need to be removed for a number of reasons including infection, non-union, fracture and revision surgery; however, removal of the Herbert screw from within healed bone can result in bone damage due to its double-threaded ends. Smooth surfaced and mechanically less secure devices such as titanium nails, Knowles pins and Kirschner wires may be easier to remove, but are prone to migration. The Echidna pin was designed to generate equivalent fixation strength, fracture compression and migration resistance compared to a Herbert screw, while facilitating ease of removal. While the findings suggest similar or superior biomechanical performance relative to that of the Herbert screw construct, future biomechanical studies ought to focus on the removal force of the Herbert screw.

There are a number of limitations of this study that ought to be considered. First, torsion of specimens may have weakened the repair construct and subsequently adversely influenced the behaviour of the construct under bending; however, since each specimen was tested in an identical manner, we do not anticipate this to significantly influence the relative differences in bending stiffness and ultimate strength between the three repair groups evaluated. Second, the cadaveric clavicle specimens harvested were embalmed and from elderly donors, and these specimens are likely to have lower strength and structural integrity compared to those from younger or fresh-frozen specimens. However, our specimens were of a similar age group, and we anticipate these effects would not significantly influence between-group differences in construct functional performance. Finally, the present study was based on a mechanical model which neglects the effects of bone remodelling and fracture healing. Therefore, conclusions about optimal fracture fixation and bone healing cannot be explicitly reported.

## Conclusion

The present study reports the biomechanical performance of a novel intramedullary Echidna pin design for mid-shaft clavicle fracture fixation. The results suggest equivalent or superior torsional and bending stability in the Echidna pin compared to that of the Herbert screw. Since the Echidna pin was developed to be stable within the intramedullary canal, maintain fracture compression and be easily removed post-operatively, it may provide a suitable alternative to conventional intramedullary screws, nails and pins; however, superior plating using bi-cortical locking screws provides substantially higher construct structural rigidity than intramedullary devices, and may therefore be useful in cases of osteoporotic bone, or where high fracture stability is required.

## Abbreviation

ANOVA: Analysis of variance
